# Reliability of the PEDro scale: comparison between trials published in predatory and non-predatory journals

**DOI:** 10.1186/s40945-022-00133-6

**Published:** 2022-03-31

**Authors:** Matteo Paci, Claudio Bianchini, Marco Baccini

**Affiliations:** 1 Unit of Functional Recovery, Azienda USL Toscana Centro, Presidio Piero Palagi, Viale Michelangiolo, 41, 50134 Florence, Italy; 2 Private Practice, Florence, Italy; 3 University of Florence and IRCCS Fondazione Don Gnocchi, Florence, Italy

**Keywords:** Randomized controlled trial, Physical Therapy Specialty, Reproducibility of Results, Periodical, Assessment

## Abstract

**Background:**

Lack of effective peer-review process of predatory journals, resulting in more ambiguity in reporting, language and incomplete descriptions of processes might have an impact on the reliability of PEDro scale. The aim of this investigation was to compare the reliability of the PEDro scale when evaluating the methodological quality of RCTs published in predatory (PJs) and non-predatory (NPJs) journals, to more confidently select interventions appropriate for application to practice.

**Methods:**

A selected sample of RCTs was independently rated by two raters randomly selected among 11 physical therapists. Reliability of each item of the PEDro scale and the total PEDro score were assessed by Cohen’s kappa statistic and percent of agreement and by Intraclass Correlation Coefficients (ICC) and the Standard Error of Measurement (SEM), respectively. The Chi-square test was used to compare the rate of agreement between PJs and NPJs.

**Results:**

A total number of 298 RCTs were assessed (119 published in NPJs). Cronbach’s alphas were .704 and .845 for trials published in PJs and NPJs, respectively. Kappa values for individual scale items ranged from .14 to .73 for PJs and from .09 to .70 for NPJs. The ICC was .537 (95% CI .425—.634) and .729 (95% CI .632-.803), and SEM was 1.055 and 0.957 for PJs and NPJs, respectively. Inter-rater reliability in discriminating between studies of moderate to high and low quality was higher for NPJs (k = .57) than for PJs (k = .28).

**Conclusions:**

Interrater reliability of PEDro score of RCTs published in PJs is lower than that of trials published in NPJs, likely also due to ambiguous language and incomplete reporting. This might make the detection of risk of bias more difficult when selecting interventions appropriate for application to practice or producing secondary literature.


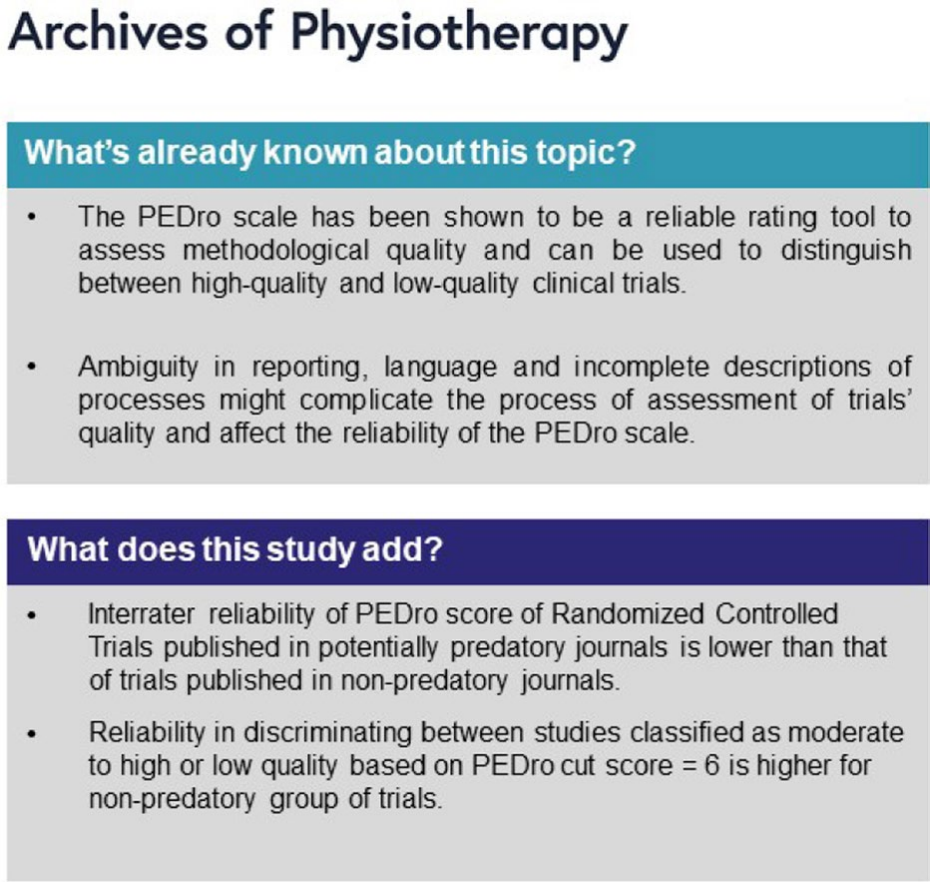


## Introduction

Randomized controlled trials (RCTs) are traditionally considered the gold standard for examining the efficacy of interventions, and the assessment of the quality of these types of studies helps to select the best clinical literature. The Physiotherapy Evidence Database (PEDro) scale is one of the most frequently used scales aimed to assess the methodological quality of RCTs in systematic reviews of interventions in physiotherapy and other fields, including medicine, nutrition and speech pathology [1]. The PEDro Scale assesses 11 items related to the study internal validity and statistical reporting, except for the first one (eligibility criteria), which is not computed in the total score. Each item is scored as either present (1) or absent (0), leading to a maximum score up to 10. A trial is considered of moderate to high quality if it scores at least 6/10 [2], though other criteria have been suggested thereafter [3]. The reliability of the scale was investigated in previous studies including trials in the field of Physiotherapy indexed in PEDro database [4, 5]. The reported ICC values ranged from 0.55 (95% confidence interval [CI]: 0.47—0.65) for the original scale [4], to 0.82 (95% CI: 0.70 – 0.89) for the Portuguese version of the Scale [5]. The reliability of the PEDro scale was also assessed in a sample of 100 RCTs included in the OTseeker, a database focused on Occupational Therapy field and modelled on PEDro [6]. The authors reported an ICC of 0.71 (95% CI 0.59 – 0.83). More recently, Yamato et al. [7] reported an ICC of 0.80 (95% CI 0.68—0.88) in a sample of trials evaluating pain medication for chronic spinal pain or osteoarthritis. A study comparing the reliability of the PEDro scale when used to assess pharmacological and nonpharmacological studies found similar values for the two fields (all studies: ICC = 0.91, 95% CI 0.83-0.94; pharmacological studies: ICC = 0.89, 95% CI 0.78-0.95; nonpharmacological studies: ICC = 0.91, 95% CI 0.84-0.952) [8]. However, among the quoted literature only Shiwa et al. [5] also estimated the measurement error, finding a SEM = 0,58 for the Portuguese version of the PEDro scale. The reliability of single items, estimated by kappa statistics, is highly variable among studies. For example, item 2 (“random allocation”) kappa ranged from of 0.13 [4] and 0.91 [5], respectively, and for item 9 (“Intention-to-treat analysis”) from 0.12 [4] and 0.91 [6]. However, kappa statistics was demonstrated to suffer from a particular paradox [9], i.e., it may assume very low values under certain conditions, even in situations of strong agreement. Thus, such different kappa values are not necessarily associated to relevant differences in the percentage of observed agreement.

Foley et al. [8] suggested that ambiguity in reporting, language and incomplete descriptions of processes might complicate the process of assessment and affect the reliability of the scale. The presence of these features might conceivably be related to the journal quality, particularly to the execution of a rigorous peer-review process of submitted articles. Although there has been much discussion about the definition of “predatory” journals [10], the term, coined by Jeffrey Beall [11], is generally related to some open access periodicals that are suspected to prioritize self-interest and accept articles for publication without proper quality checks, collecting large amounts of money in author’s fees [12]. Indeed, this concern is supported by consistent recent findings showing that predatory journals (PJs), i.e., journals included in the Beall's list, have significantly shorter peer review processes than non-predatory journals (NPJs) [13–15]. We hypothesized that this shortcoming should increase the occurrence of ambiguity or incompleteness in methodological reporting, resulting in poor reliability of the PEDro scale in addition to making readers less confident in selecting interventions appropriate for application to practice. Thus, the aim of this investigation was to compare the reliability of the PEDro scale when the scale is applied to assess the quality of physiotherapy trials published in PJs and NPJs.

## Methods

### Identifying eligible journals and trials

This is a secondary analysis of a previous investigation aimed to compare the methodological quality of RCTs published in PJs and in NPJs in the field of physiotherapy [15]. A list of 18 physiotherapy journals, which were active during the period 2014–2017 and included either in the Beall list (*n* = 9) or in the Directory of Open Access Journals (DOAJ), were selected. A detailed description of journals and trials selection process is available elsewhere [15].

A total number of 410 RCTs were assessed using the PEDro scale. For 112 trials, the PEDro score was extracted from the PEDro database. The remaining 298 trials (179 and 119 from PJs and NPJs, respectively) were independently rated by two raters randomly selected among a convenience sample of 11 assessors. Six of them were physiotherapists (1 PhD, 5 MSc) certified as “PEDro friends”, since they had passed the test after the training package of PEDro (available at: https://training.pedro.org.au/). The others (one student from the Bachelor in Physiotherapy, one physiotherapist attending the Master in Rehabilitation Sciences for Health Professions, and three MSc physiotherapists with more than 20 years of experience in research) were trained with examples and practice papers by the certified raters. Each rater independently rated with the PEDro scale a variable number of RCTs (44 to 77) randomly assigned. Any disagreement was resolved by a randomly selected third rater, who was informed only about items that generated disagreement and required his/her judgement. Each rater was blinded to the evaluation of the other raters. The assessors’ randomisation was carried out using an online tool (www.randomized.org).

### Statistical analysis

All inter-rater reliability indexes were computed including all articles and separately grouping the trials in two categories, i.e., trials published in NPJs, or in PJs. Cronbach’s alpha was calculated for internal consistency, considering values from 0.70 to 0.95 as acceptable [16]. The inter-rater reliability of dichotomous judgments for each item was evaluated with the Cohen’s kappa statistic and agreement interpreted as suggested by Landis and Koch [17]: kappa = 0 = none; 0.01-0.20 = none to slight; 0.21-0.40 = fair; 0.41-0.60 = moderate; = 0.61-0.80 = substantial, and 0.81–1.00 = almost perfect. Since Cohen’s kappa might be affected by a paradoxical behavior, agreement on the presence of the item (P + +), symmetry expressions for agreement (Sa) and disagreement (Sd) were also reported, as recommended by Lantz and Nebenzahl [18]. For each item, the rate of agreement was compared between trials published in NPJs, or in PJs using the Chi-square test.

The inter-rater reliability of the total PEDro score was evaluated by computing type 1,1 Intraclass Correlation Coefficients (ICC_1,1_) with 95% confidence intervals (CI). ICC values were interpreted according to the guidelines of Fitzpatrick et al. [19], i.e., ICC = 0.70 and ICC = 0.90 should be considered the minimum acceptable levels for measures to be used when assessing groups (in this case, groups of trials) or individuals (single trials), respectively. The guidelines suggested by Fleiss [20] (i.e., values less than 0.40 = poor reliability; values between 0.40 and 0.75 = good reliability; values greater than 0.75 = excellent reliability) were also considered to compare results to previous studies, since they all used that reference. However, the more restrictive guidelines have been recently recommended as more appropriate [21, 22]. The Standard Error of the Measurement (SEM) of total PEDro scores was also calculated using the formula: SEM = SD*√(1-ICC) [23]. Finally, the inter-rater reliability of PEDro scale in discriminating between trials which were classified as high-quality or low-quality studies using a score > 5 as cut-off [4] was also estimated with the Cohen’s kappa statistic. Data analyses were performed using the SPSS statistical package 20.0 for Windows.

## Results

A total number of 298 RCTs were assessed using the PEDro scale (119 published in NPJs). Table 1 shows the reliability indexes computed in the whole sample of trials and Table 2 shows the same indexes computed separately in non-predatory and predatory trials.

**Table 1:** Reliability of the PEDro scale assessed in the whole sample of trials

PEDro score	ICC (95% C.I.)				SEM		
	0.639 (0.566–0.701)				0.795		
	**% agreement**	***k***	**interpretation**	**P + + **	**Sa**		**Sd**
**PEDro scale item**							
2 Random allocation	95.0	.01	none to slight	94.6	0.99	0.03	
3 Concealed allocation	91.6	.71	substantial	13.1	-0.71	0.00	
4 Groups similar at baseline	69.5	.36	fair	45.0	0.29	0.02	
5 Subject blinding	93.0	.65	substantial	7.7	-0.83	0.03	
6 Therapist blinding	97.3	.59	moderate	2.0	-0.96	0.00	
7 Assessor blinding	88.6	.65	substantial	15.1	-0.66	0.04	
8 Less than 15% dropouts	64.8	.30	fair	36.6	0.13	-0.20	
9 Intention-to-treat analysis	59.7	.15	none to slight	17.1	-0.43	-0.21	
10 Between-group statistical comparisons	87.9	.39	fair	82.9	0.88	0.07	
11 Point measures and variability data	92.6	.55	moderate	87.2	0.88	0.01	
**Quality discrimination**^**a**^	73.5	.41	moderate	21.1	-0.42	-0.05	

^*a*^*Based on PEDro cut score* = *6 (see main text for details)*

*ICC* Intraclass Correlation Coefficient, *CI* confidence intervals, *SEM* Standard Error of the Measurement, *P* +  + agreement on the presence of the item, *Sa* symmetry expressions for agreement, *Sd* symmetry expressions for disagreement

**Table 2:** Reliability of the PEDro scale estimated in trials published in non-Beall and in Beall journals

PEDro score	non-Beall journals						Beall journals					
	**ICC (95% C.I.)**				**SEM**		**ICC (95% C.I.)**				**SEM**	
	0.729 (0.632-0.803)				0.957		0.537 (0.425-0.634)				1.055	
	**% agreement**	**K**	**interpretation**	**P + + **	**Sa**	**Sd**	**% agreement**	**K**	**interpretation**	**P + + **	**Sa**	**Sd**
**PEDro scale item**												
2 Random allocation	95.8	N/a		95.8	1	0.04	94.4	.14	none to slight	93.8	0.99	0.02
3 Concealed allocation	89.1	.68	substantial	16.0	-0.64	0.05	93.3	.73	substantial	11.2	-0.76	-0.02
4 Groups similar at baseline	81.5	.62	substantial	48.7	0.20	-0.08	61.4	.19	none to slight	42.5	0.38	0.12
5 Subject blinding	90.8	.68	substantial	12.6	-0.72	0.05	94.4	.59	moderate	4.5	-0.90	0.02
6 Therapist blinding	95.8	.59	moderate	3.36	-0.93	-0.03	98.3	.56	moderate	1.1	-0.98	0.02
7 Assessor blinding	88.2	.70	substantial	21.0	-0.52	0.00	88.8	.60	moderate	11.2	-0.75	0.06
8 Less than 15% dropouts	71.4	.42	moderate	42.9	0.20	-0.02	60.3	.24	fair	32.4	0.07	-0.34
9 Intention-to-treat analysis	60.5	.21	fair	26.0	-0.14	0.18	59.2	.14	none to slight	11.2	-0.62	-0.49
10 Between-group statistical comparisons	89.1	.09	none to slight	88.2	0.98	0.04	87.1	.48	moderate	79.3	0.82	0.07
11 Point measures and variability data	89.9	.40	fair	85.7	0.91	**0.03**	94.4	.66	substantial	88.3	0.87	0.04
**Quality discrimination**^**a**^	79.0	.57	moderate	31.9	-0.19	0.07	69.8	.28	fair	14.0	-0.60	-0.14

*N/a Not available since no Table **2* × *2 created*

^*a*^*Based on PEDro cut score* = *6 (see main text for details)*

*ICC* Intraclass Correlation Coefficient, *CI* confidence intervals, *SEM* Standard Error of the Measurement *P* +  + agreement on the presence of the item, *Sa* symmetry expressions for agreement, *Sd* symmetry expressions for disagreement

For the whole sample, Cronbach’s alpha was 0.779; the ICC was 0.639 (95% CI: 0.566—0.701) and the SEM 0.795; kappa values ranged from 0.01 to 0.71. For trials published in PJs and NPJs, Cronbach’s alpha was 0.704 and 0.845, respectively. The inter-rater reliability of the PEDro score was lower for trial published in PJs (ICC: 0.537, 95% CI: 0.425—0.634; SEM: 1.055) than for trials published in NPJs (ICC: 0.729, 95% CI: 0.632—0.803; SEM: 0,957). Kappa values for individual items ranged from 0.14 to 0.73 in PJs and from 0.09 to 0.70 in NPJs. The difference in the frequency of agreement between PJs and NPJs was highly significant (*p* < 0.001) for item 4 (“Groups similar at baseline”) and barely significant (*p* = 0.050) for item 8 (“Less than 15% dropouts”). In both cases, the agreement was found more frequently in the trials published in NPJs than in the trials published in PJs.

The inter-rater reliability in discriminating between studies of high and low quality, evaluated with the kappa statistic, was 0.41 (percentage of agreement 73.5%) for the whole sample of articles, 0.28 (percentage of agreement 69.8%) and 0.57 (percentage of agreement 79.0%) for trials published in PJs and NPJs, respectively.

## Discussion

We will discuss our findings in light of data reported by Maher et al. [4], Shiwa et al. [5] and Tooth et al. [6], since they are the only published articles that evaluated the PEDro scale reliability in trials published in the field of Physiotherapy [4, 5] and in the similar field of Occupational Therapy [6]. To make any comparison easier, the findings of the present study and of the published literature are summarized in Table 3. The ICC of the PEDro score found in the present study, when including the whole sample of articles, is slightly higher than the value found by Maher et al. [4] for individual ratings, but lower than values found by Shiwa et al. [5] and Tooth et al. [6]. The standard error of the measurement of the scale was nearly double than previously reported by Shiwa et al. [5], also due to the higher variance of the PEDro score of the trials assessed. Indeed, we included a far larger sample of trials that covered the full range of PEDro score (0–9), whereas the articles assessed by Shiwa et al. [5] scored 1–7 at the PEDro scale.

**Table 3:** Inter-rater reliability indexes (PEDro scale items: Cohen’s kappa; PEDro score: Intraclass Correlation Coefficient, ICC) found in the present study and in the published literature

	Present study^a^			Maher, 2003^a^	Maher, 2003^b^	Shiwa, 2011^b^	Tooth, 2005^a^
	PJs	NPJs	All				
Item 2	.14	NA	.01	.13		.91	NA
Item 3	.73	.68	.71	.62		.73	.87
Item 4	.19	.62	.36	.40		.60	.53
Item 5	.57	.68	.65	.66		1.00	NA
Item 6	.56	.59	.59	.33		1.00	NA
Item 7	.60	.70	.65	.73		.78	.86
Item 8	.24	.42	.30	.42		.53	.56
Item 9	.14	.21	.15	.12		.66	.88
Item 10	.48	.09	.39	.62		.66	NA
Item 11	.66	.40	.55	.59		.74	.55
ICC (95% CI)	.54 (.43-.63)	.73 (.63-.80)	.64 (.57 -.70)	.55 (.41-.72)	.68 (.57- .76)	.82 (.70-.89)	.71 (.59-.83)

*PJs* predatory journals, *NPJs* non-predatory journals

^a^*Individual rating, *^*b*^*consensus rating*

As for the reliability of dichotomous ratings of individual PEDro scale items, it needs to be interpreted also considering the distribution of data within the contingency matrix in addition to kappa statistics. As pointed out in the introduction, in fact, an unbalanced distribution may lead to low kappa coefficients even when the observed agreement is near to 100% [9, 18]. In the present study, the items 4 (“Groups similar at baseline”), 8 (“Less than 15% dropouts”) and 9 (“Intention-to-treat analysis”) showed the lowest values in terms of either kappa coefficient and percentage of observed agreement, so they have poor reliability. For item 4 and 8, this result was also reported by previous investigations [4, 6]. Maher et al. [4] found none to slight agreement also for item 9, as indicated by kappa coefficient, in the face of relatively high observed agreement. Conversely, this item showed almost perfect agreement in the study of Tooth et al. [6], in terms of both kappa statistics and observed agreement. It is noteworthy that also in the analysis of Shiwa et al. [5] all the three items showed the smallest kappa coefficients. Though Shiwa et al. [5] do not report the total observed agreement, they provide the percentage of agreement on the presence of the items, that is similar to what was found in the other studies. Thus, we can speculate that even when a consensus rating is conducted, the agreement on these items may be low.

Likely, the poor reliability of these items can occur because they required more judgment and higher skill than the others. For “Groups similar at baseline” raters need to judge whether any baseline differences among groups might affect outcomes; however, authors frequently report data of baseline assessment (e.g., means and SD) without providing any statistical comparisons, which would help raters to decide whether any differences actually exist. “Less than 15% dropouts” seems far easier to rate, but often authors provide information about dropouts in tables rather than in the main text of the article, or do not provide that information at all. “Intention-to-treat analysis” is generally a poorly understood term [24, 25]. According to Fisher et al. [26], the intention-to-treat analysis includes every subject who is randomized according to randomized treatment assignment, regardless of anything that happens after randomization, including withdrawal. Thus, it is theoretically linked to missing data due to dropouts, because an ideal intention-to-treat analysis would require follow-ups on all participants [27–29]. However, the PEDro scale separates loss to follow-up (item 8) from intention-to-treat analysis (item 9), so it is possible to rate item 9 as satisfied (i.e., an intention-to-treat analysis was conducted) when there is incomplete follow-up and the authors decide not to impute the missing data [30]. We may speculate that strict raters might score item 9 as unsatisfied even when the authors state they did analyze the data per intention-to-treat but excluded dropouts from analyses.

The main finding of our study is that the reliability of the PEDro scale total score is lower when applied to assess trials published in PJs than trials published in NPJs. For the former, the ICC exceeded the minimum acceptable value for assessment at a group level, according to Fitzpatrick et al. [19], whereas for the latter also the upper limit of the 95% CI of the ICC was under that value. Indeed, the upper limit of ICC 95% CI found in PJs is quite the same as the lower limit found in NPJs, so we can speculate that the difference is barely significant at the 0,05 level. It is noteworthy that according to the guidelines of Fleiss [20], the reliability of the total score would always be classified as good, independently from the samples of trials included (predatory, non-predatory and the whole sample). Despite the different ICC, the measurement error approximates 1 for both articles published in NPJs and PJs, being only slightly smaller in the former. This can be explained by the higher variance of the PEDro scores of the articles published in NPJs compared to PJs (SD = 1.84 and 1.55, respectively).

Kappa values and percentages of agreement for PEDro scale items are more often lower in trials published in PJs than in trials published in NPJs. However, differences are generally low except for item 4 (“Groups similar at baseline”) and, to a lesser extent, for item 8 (“Less than 15% dropouts”). It is noteworthy that these are the only items where the difference between the two categories of journals were significant. Conversely, kappa values were higher for trials published in PJs compared to those published in NPJs for item 11 (“point measures and variability data”) and even more for item 10 (“Between-groups statistical comparison”). In both cases, however, the percentage of agreement was quite similar for articles published in PJs and NPJs, as confirmed by the Chi-square test; thus, the differences in kappa values do not reflect a different agreement between the raters and seem to be related to the above-mentioned “kappa paradox [9, 18]. Indeed, for item 10 both the base rate for ‘yes’ response (i.e., both raters agreed it was present) and the asymmetry in agreement are higher in trials published in NPJs compared to those published in PJs, resulting in lower kappa value even if the percentage of agreement in slightly higher. The effect of an imbalanced data distribution is clear also for item 2 where the kappa coefficient indicates “none to slight agreement” but the percentages of agreement between raters are about 95%. Kappa coefficient for item 2 was not available for trials published in NPJs since one rater always scored the item as present, but we may be confident that otherwise it would have been very low. Excluding item 2, more items showed substantial agreement in NPJs (*N* = 4) than in PJs (*N* = 2), and more items showed none-to-slight agreement in PJs (*N* = 2) than in NPJs (*N* = 1).

Reliability in discriminating between studies classified as moderate to high or low quality based on PEDro cut score = 6 is also higher for group of trials published in NPJs. This issue is relevant due to the increasing number of systematic reviews that used the PEDro scale to rate the quality of included trials [1], possibly adopting the cutoff to distinguish moderate to high quality from low-quality studies.

One possible explanation of the different values found for trials published in PJs and NPJs is related to the article quality of reporting. Indeed, the interpretation of the strengths and limitations of an RCT relies on clear reporting of trial methodology [31], which in turn might depend on the experience of the authors and on the quality of the peer reviewing process. The sample size of included trials and the type of PEDro rating (individual, i.e., rating made by a single rater, or consensus, i.e., rating made by a panel of 2 or 3 raters) might also greatly impact the results. The year of publication might also have indirect impact on results since the reported methodological quality has improved over time [32].

Among the articles selected in the present study, those published in PJs largely outnumber those published in NPJs. A lack of a robust peer review process and editorial services seems to be a key feature of PJs [33], and authors of papers published in these journals were found to be largely inexperienced researchers who did not publish any articles previously [34]. A combination of these two features might contribute to ambiguous language, incomplete descriptions of the procedures in the Method section and/or erroneous positioning of methodological details in the Result or Discussion sections. All these features might complicate the rater's decision about the satisfaction of PEDro criteria and increase the chance of disagreements between raters [8].

Both Maher et al. [4] and Shiwa et al. [5] assessed trials indexed on the PEDro database, which includes (in addition to guidelines and systematic reviews) only RCTs studying the effect of physiotherapy interventions, independently from the journal of publication. Thus, a journal does not need to be indexed in PubMed or other databases of scientific publications so that its articles are included and scored on PEDro. Although Maher et al. [4] conducted their research in a period when the phenomenon of PJs was just at the beginning, we may hypothesize that also some of the RCTs selected in that study (all but one published in the 1980s and 1990s) were obtained from journals that were not used to conducting a rigorous peer-review process, as the majority of the articles included in the present study are suspected to be. However, the number of RCTs assessed by Maher et al. [4] is very low (*N* = 25), and it is recognized that sample size needs to be large enough to produce sufficiently accurate reliability estimates [35]. Shiwa et al. [5] assessed a higher number of articles (*N* = 50) but selected only articles written in Portuguese. We searched for Portuguese-language RCTs included in the PEDro database up to 2 August 2010 (date when articles were downloaded from PEDro by the authors) and we found that none of them were published in journals included in the Beall’s list. If the reliability of the scale is also affected by the journal quality, as found in the present study, the absence of RCTs published in PJs might partly explain the better results found in that study. Moreover, Shiwa et al. [5] reported the reliability of consensus rating, rather than individual rating. As shown by Maher et al. [4], the consensus rating of the PEDro total score is more reliable than individual rating. Indeed, the ICC values reported by Shiwa et al. [5] are the highest among all the studies that assessed the reliability of the PEDro scale applied to trials in the field of Physiotherapy or Occupational Therapy. The lack of RCTs published in PJs within the sample of trials selected might explain also the higher ICCs found by Tooth et al. [6] compared to the present study. We ascertained that also in the OTseeker database no articles published in PJs were indexed up to the date (May 2003) when Tooth et al. randomly selected the sample of 100 RCTs included in their study. Interestingly, the ICC found by Tooth et al. [6] are quite similar to the ICC found in the present study when only NPJs were selected.

### Limitations

Some methodological biases might have influenced the results. First, most of our raters were a mixed group of physiotherapists who were formally or not formally trained in the use of the scale; thus, the different expertise might have contributed to some extent to the observed disagreement. However, we may speculate that the effect should have been minor, if any, because the reliability of the scale was found similar in studies that involved formally trained raters [4–6] or raters who were familiar with the PEDro tool and expert in research methodology but did not attend any formal training [7, 8]. Most importantly, raters were not blind to the source or the articles assessed, so we cannot assure their blinding to the inclusion of journals in the Beall’s list. Though they were not directly informed about this feature, and were discouraged to explore it, we could not remove any references to the journal from each article, so raters actually were able to retrieve this information. Knowing if articles under rating were from a predatory or a non-predatory publisher might have influenced their assessment*.* Lastly, criteria for classifying journals as PJs or NPJs are not fully defined; several lists exist, in addition to the Beall’s one, to help identify the former, but they are not consistent [12]. Similarly, being listed in the DOAJ does not guarantee the journal quality.

Indeed, some journals are included both in DOAJ and Beall’s lists, and this was the case also for one journal classified as PJ in the present study [15]. We may speculate that errors in classifying the included journals as PJs or NPJs might have reduced the observed differences between them.

The sample size was not a priori calculated for this study, because it depended on the number of included journals and trials and on the number of trials not rated in the PEDro database [15]. However, the number of included trials seems to be appropriate and would be rated as “very good” (i.e., ≥ 100 in both NPJs and in PJs) according to COSMIN Study Design checklist [36].

## Conclusions

Interrater reliability of PEDro score of RCTs published in PJs is lower than that of trials published in NP**J**s. Ambiguous language and incomplete reporting might be one relevant and specific source of lower reliability and make a thorough detection of risk of bias more difficult. This finding should be considered when selecting interventions appropriate for application to practice and when evaluating clinical trials to produce secondary literature.

## Data Availability

All data generated or analysed during this study are included in this published article.

## Abbreviations

RCT: Randomized Controlled Trial
PJ: Predatory journal
NPJ: Non-predatory journal
ICC: Intraclass Correlation Coefficient
SEM: Standard Error of the Measurement
